# Quantum Chemical Stability Analysis of Phthalocyanine Metal One-Dimensional Polymers with Bidentate Ligands

**DOI:** 10.3390/molecules29174111

**Published:** 2024-08-30

**Authors:** Anna Szwajca, Radosław Pankiewicz

**Affiliations:** Faculty of Chemistry, Adam Mickiewicz University in Poznań, Uniwersytetu Poznańskiego 8, 61-614 Poznan, Poland; aniasz@amu.edu.pl

**Keywords:** phthalocyanine zinc complexes, coordination polymer, N-donor ligands, molecular prediction, DFT calculation

## Abstract

The combination of metal–phthalocyanine complexes and axially coordinated organic molecules into polymer chains presents a significant challenge in the synthesis of hybrid materials. A calculated structure for one-dimensional coordinate polymers with N-donor ligands using ab initio (PM6) and DFT (LanL2Dz) methods is presented. DFT methods have shown that there is a linear, one-dimensional structure without distorted geometry for the two bipyridine ligands. The components of the proposed polymers consist of square-planar Zn complexes of phthalocyanine (PcZn) connected via bridging ligands (L). Electronic properties of the monomer PcZnL of zinc phthalocyanine with bidentate ligands have been analyzed using calculations based on density functional theory (B3LYP6-31G(d,p)). Molecular orbital calculations show that this connection between the metallomacrocycle and the conjugated ligand results in a small energy gap, promising molecularly active materials as conductors. The crystallographic reports indicate that obtaining this kind of polymer with the participation of Pc Zn and bidentate ligands is possible.

## 1. Introduction

Phthalocyanine-shaped molecules are P-type semiconductors characterized by high thermal and chemical stability. They also exhibit interesting optical, electrical, and magnetic properties with good prospects in electronics, optoelectronics, and photovoltaics [1,2,3,4]. Phthalocyanine (Pc) molecules, with their ability to incorporate various metal ions at their center, can easily tune the electronic and optical properties of the compound without significantly altering its overall structure [5,6]. Metal complexes containing phthalocyanine allow for self-assembly on metal surfaces without aggregation. These macrocyclic compounds have also shown interesting molecular switching behaviors [7]. A significant disadvantage of these compounds is their low solubility. To improve the solubility of metallomacrocycle compounds, the macrocyclic ring can be modified by substituting hydrogen atoms with various organic substituents or additional metal center ligation. Modifying the solubility of phthalocyanines by coordinating additional axial ligands to the metal center is an effective, one-step strategy. This approach can significantly alter the physical and chemical properties of phthalocyanine, including its solubility and reactivity. Limiting the considerations to one central atom allowed for a significant focus in the planned research. The titular PcZn has unique properties that make it a valuable tool in photodynamic therapy and other areas of medicine and technology. Its ability to deeply penetrate tissues, high efficiency of ROS generation, and versatility in various applications make it unique compared to other phthalocyanines. Numerous publications in the literature describe the stability of PcZn complexes with N-donor ligands, the interpretation of UV-Vis-NIR spectra of modified PcZn, the characteristics of hydrogen bonds in dimeric zinc phthalocyanine complexes, and the effect of the solvent on the planarity and aromaticity of free and monohydrate zinc phthalocyanine [8,9,10,11]. Our attention was also drawn to publications documenting the crystallographic structures of the Zn complex and studies in the literature on methods of polymer synthesis involving phthalocyanines [3,10,11,12]. Both crystallographic results and synthetic reports influenced the concept of our research. When planning polymers with Pc Zn, the combination of Pc with a bidentate ligand in a 1:1 ratio should be considered as the main motif of the coordination polymer. Known structures of 1:1 PcZn complexes included bidentate N-donating ligands, such as pyrazine, imidazole, N-methylimidazole, and N-(2 pyrimidinyl) imidazole, which were obtained in crystalline forms [13,14,15,16,17,18,19]. Such complexes can be obtained in the reaction carried out in an autoclave at high temperatures and for several days [14]. Data indicate that most examples of Pc Zn coordination complexes with bidentate ligands are formed in an H shape(Pc/ligand/Pc) and T shape (Pc/ligand) [14,15]. This is a temperature-dependent reaction. The 2PcZn/1ligand complex is more stable than the 1PcZn/1ligand complex. The axial bond of the Zn and N atoms of ligand in the T-shaped complex is stronger by about 0.029 Å than in the H-shaped complex. These results demonstrate that the bridging effect of Pc rings with ligands is achievable and controllable. Axial complexes show better solubility than the initial Pc; thus, it is easier to carry out subsequent stages of polymer band growth. PcZn complex crystals are soluble in 1-chloronaphthalene, quinoline, pyridine, and other N-donating solvents and also slightly soluble in benzene, DMSO, and CH_2_Cl_2_. The strategy of polymer compounds seems to be effective in obtaining crystalline and highly soluble polymers [14].

## 2. Results and Discussion

**Figure 1:**
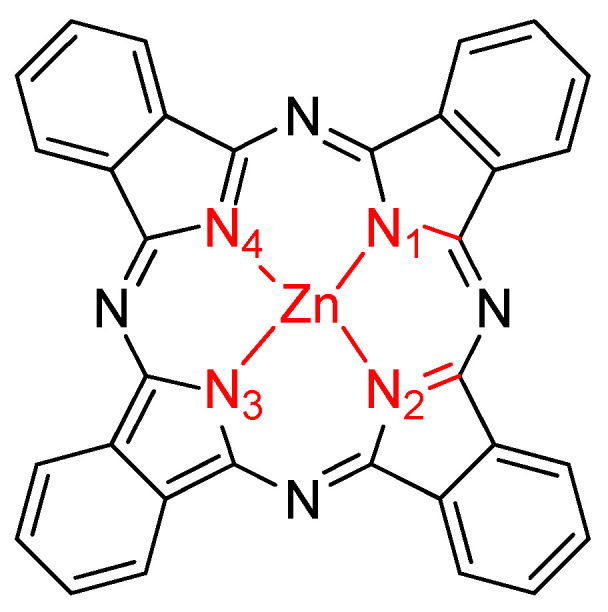
Structures and numbering of PcZn.

In the center, the phthalocyanine PcZn molecule contains four pyrrole subunits connected by four azomethine bridges, representing the porphyrinoid class, and forms a complex macrocycle (Figure 1). In our structural considerations, we limited ourselves to examining the connections between the central Zn atom and the four nitrogen atoms.

**Figure 2:**
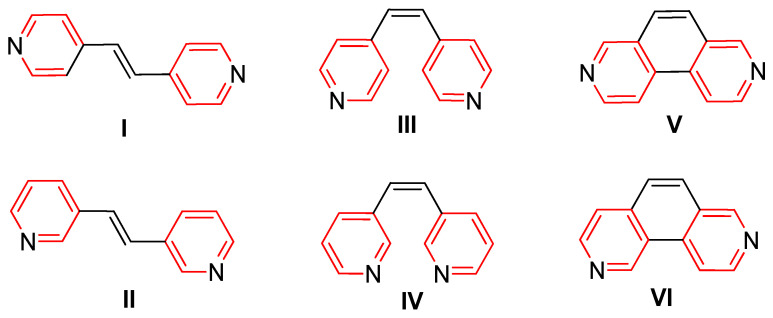
Pyridine-based bridged ligands (L1-LVIs), where π-conjugated bridges connect the pyridine rings.

The N-heterocycles selected for the study can be used as ligands in coordination and organometallic chemistry [20,21,22,23]. All of the compounds (Figure 2 I–VI): (*E*)-1,2-di(pyridin-4-yl)ethane (I), (*E*)-1,2-di(pyridin-3-yl)ethane (II), (*Z*)-1,2-di(pyridin-4-yl)ethane (III), (*Z*)-1,2-di(pyridin-3-yl)ethane (IV), 3,8-phenanthroline (V), and 2,8-phenanthroline (VI) are commercially available compounds or their laboratory synthesis results in a pure product. The basic structural element of these compounds is two pyridine rings connected in various ways by the -CH_2_=CH_2_- group. The degree of complexity of such compounds ranges from alkyl-separated pyridines to phenanthroline systems. All of them are bidentate, conjugated linkers between zinc atoms.

**Figure 3:**
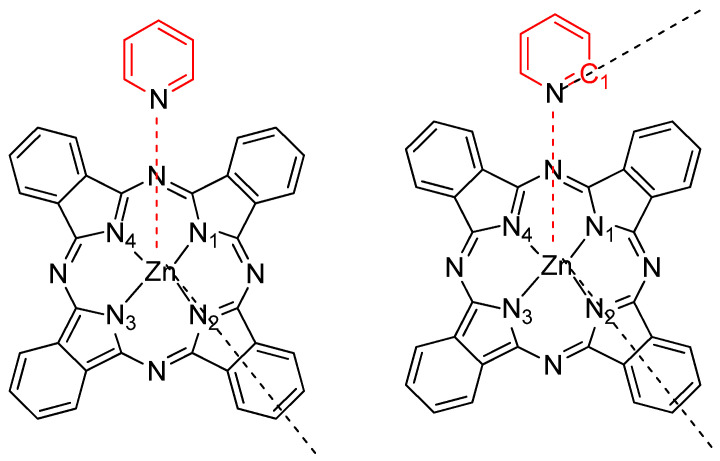
Pyridine-based ligands in coordination complex with PcZn.

The crystal structures of axially coordinated PcZn complexes, known from the literature, such as PcZn(4-CH_3_Py), PcZn(4-NH_2_Py), PcZn(1,4-diPy) and 1,2-di(4-Py)ethane, 1,2-di(4-Py) dyl)ethylene, 1,2-di(4-Pypropane), where the central Zn atom is coordinated by four nitrogen atoms from isoindole units of the Pc ring system and one nitrogen atom from the pyridine ligand, provide detailed information on bond lengths. The bond distances from the Zn atom to the N1-N4 atoms of the Pc ring range from 2.0278 to 2.03 Å, while the distance to the nitrogen atom of the axial ligand is slightly longer, at 2.09 Å. The angle between the Pc plane (referred to as the N4 plane) and the plane of the PcZn(4-NH_2_Py) molecule is 87.60°, indicating that the pyridine-based ligand is almost perpendicular to the Pc plane [13,18,24,25]. All bond distances and angle relationships were included in the calculated structures. In addition to the angle between the axial ligand and the phthalocyanine ring plane, the dihedral angle was also analyzed, as shown below (Figure 3).

The assumption that the appropriate linker for phthalocyanine rings would be a bridging ligand consisting of two pyridine rings was confirmed by the obtained calculations. Among the selected structures of bidentate ligands, the distance between nitrogen atoms varied, from the shortest at 6.53 Å for ligand L VI to the longest at 9.49 Å for ligand LI, which corresponds to the intended distance between Pc rings in the target polymers.

### 2.1. Geometry Optimization of PcZn Complex with Ligands (I–VI)

**Figure 4:**
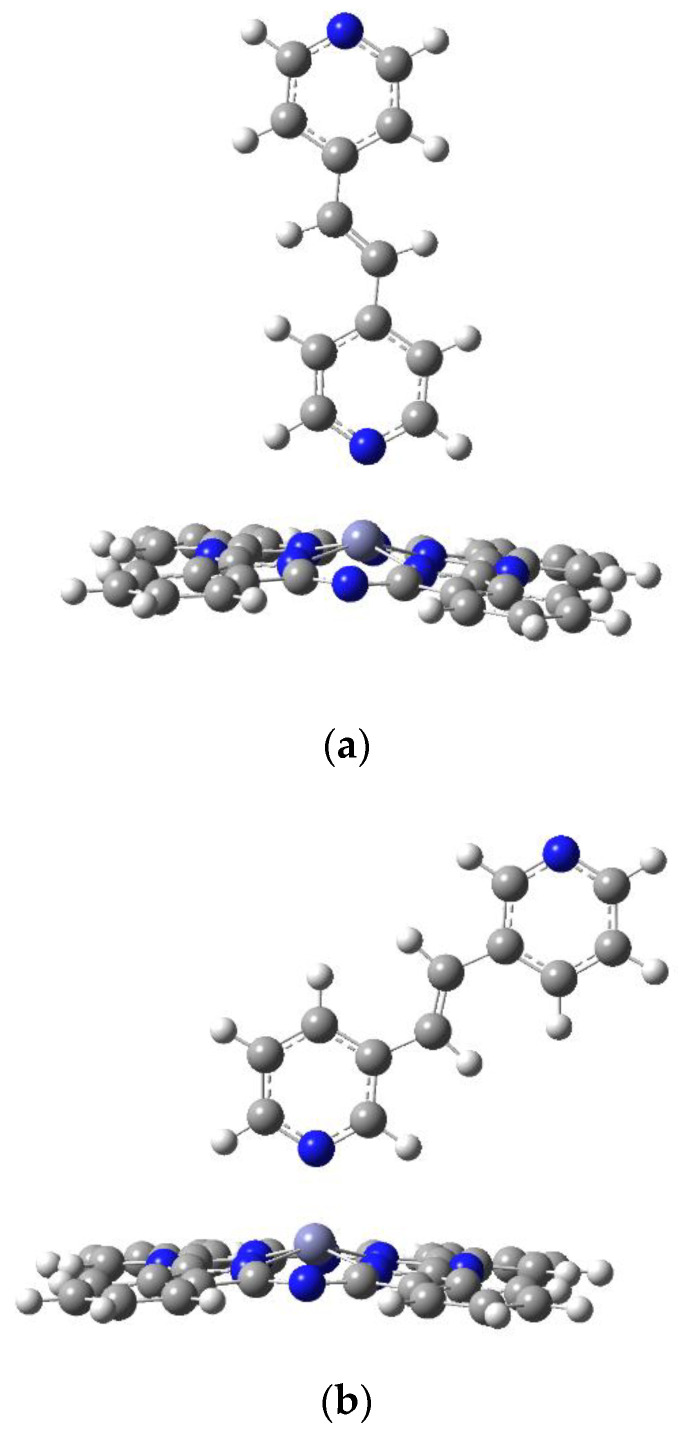
The optimized geometries for the model Zn phthalocyanines complex with ligands LI (**a**) and LII (**b**), optimized at the B3LYP/6-31G(d,p). Color code of atoms: C—dark grey, H—light grey, N—blue.

The optimization of PcZn coordination complexes was performed using the DFT/B3LYP method with the 6-31G(d,p) basis set. The XYZ coordinates of the calculated structures are provided in the Supplementary Materials. For the PcZn polymers, the LanL2DZ basis set was employed. For theoretical calculations of macrocyclic electronic structures, modern density functional theory (DFT) approaches are currently most suitable for accuracy and computational cost. The DFT calculations performed in this work were utilized based on their sufficient accuracy with minimal computational cost. For the DFT calculations performed on the complex of Zn phthalocyanines, the medium-sized 6-31G(d,p) was used. Calculations on similar systems with larger basis sets provided similar accuracies to 6-31G(d,p) [9]. The calculated geometric parameters of the proposed axial complexes of zinc phthalocyanine with bipyridine derivatives as bidentate ligands are listed in Table 1. In LI-LVI ligand, both pyridine rings are conjugated, resulting in four planar structures and two non-planar structures. The geometric arrangement of the ligand relative to the Pc ring is consistent in each case, regardless of the complexity of the ligand (Figure 4a,b). The obtained bond lengths between the central zinc atom and the nearest four nitrogen atoms (2.04 Å) closely match the bonds measured in crystallographic structures, which are 2.03 Å.

As expected, the Zn–N ligand connection is slightly longer than the others, measuring 2.15 Å compared to 2.10 Å in crystallographic systems. The convergence of the N ligand Zn-N angle of the Pc ring (on average about 100°) and the dihedral angle (43–45°) confirms that the model systems accurately reflect the real structure for ligands containing a pyridine ring as a direct link with the phthalocyanine metal.

A significant diversity among the selected group of ligands was observed when comparing their calculated stability energy values (Table 2). The favorable systems were the PcZn complexes with the LI and LIII ligands, followed by LII, LIV, and LVI. The least stable system was PcZnLV. These values indicate the complexity of matching the axial ligand with the phthalocyanine ring. The Zn-NPy distance suggests that the bond is of a coordinate nature, and the negative charges are delocalized evenly on the Pc ring atoms, which provides additional stabilization.

**Figure 5:**
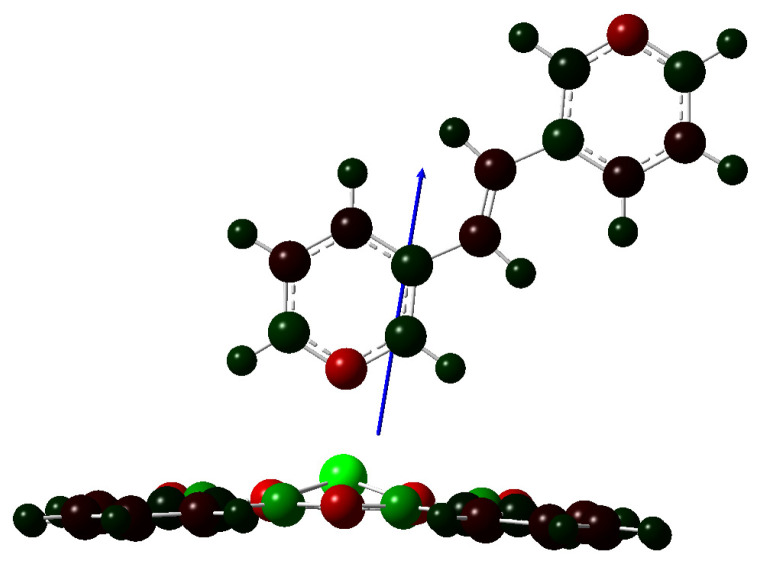
Molecular structure of PcZnII: optimized geometry calculated at the level of DFT/B3LYP/6-31G (d,p) theory with dipole moment orientation (4.5939Debye) and distribution of partial atomic charges (Mulliken) from −0.991 (red color) to 0.991 (green color).

The coordination complex PcZnII is the main motif of the coordination polymer Pc(PcZnII)_3_. The optimized ligand geometry is shown in Figure 5, along with the dipole moment orientation and partial atomic charge distribution (Mulliken). Comparing the data ligand vs. complex, one can see the change in the dipole moment orientation and its value, i.e., from 0 Debye (in the ligand) to 4.5939D Debye (in the complex).

### 2.2. HOMO LUMO of PcZn Complex with Ligands (I–VI)

Individual variability in the studied group of ligands is also reflected in the calculated energy gaps of the HOMO and LUMO molecular orbitals (Table 3). These values allowed us to characterize the selected group of complex compounds for their kinetic stability and chemical reactivity, which was similar to the values of the band gaps of Pc metal with bidentate ligands known from the literature [26]. The system with the smallest energy difference between the HOMO and LUMO orbitals was the PcZnLI compound (Eg = 1.8732 eV).

**Figure 6:**
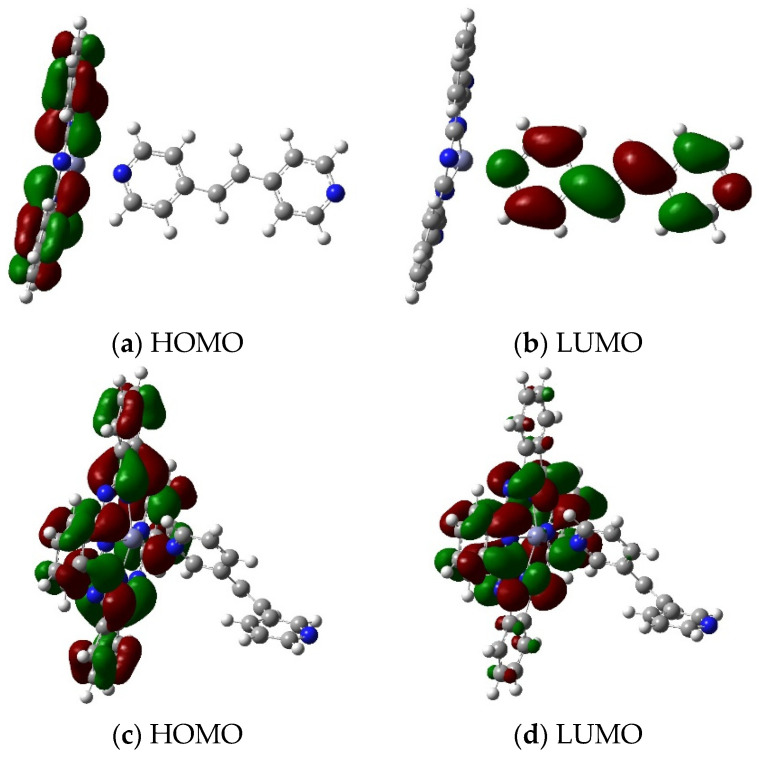
PcZnLI (**a**,**b**) and PcZnLIV (**c**,**d**) HOMO and LUMO orbitals obtained with B3LYP/6-31G(d,p). HOMO and LUMO orbitals obtained with B3LYP/6-31G(d,p). Red and green colors indicate the different phases of the molecular orbitals.

Figure 6a,b show a three-dimensional graph of the calculated molecular orbitals (B3LYP/6-31G(d,p)) for the PcZnLI complex. In the HOMO and LUMO diagrams, red and green colors indicate the different phases of the molecular orbitals. These phases are crucial for understanding constructive or destructive interference during chemical reactions. Below the drawing of the HOMO/LUMO orbital system for the compound with the greatest application potential, PcZnLI, the values and graphical representation of the orbitals for the PcZnLIV compound (Figure 6c,d), which has the lowest parameters in the given group, are displayed.

The theoretical planning of the range of Pc-type complexes with axial ligands reveals the complexity of matching the structure to the expected applications. Among the presented group, the phthalocyanine zinc (PcZn) with the axial ligand LI is the most energetically stable and exhibits good optical properties. The HOMO orbital is mainly distributed over the system of the Pc fragment. In turn, the unoccupied LUMO orbital is distributed over the ligand fragment (Figure 6a,b). A different distribution is observed for PcZnLIV, where the HOMO and LUMO orbitals are localized in the Pc fragment.

### 2.3. Geometry Optimization of Polymers Pc(PcZnL)_3_ (L = I and II)

**Figure 7:**
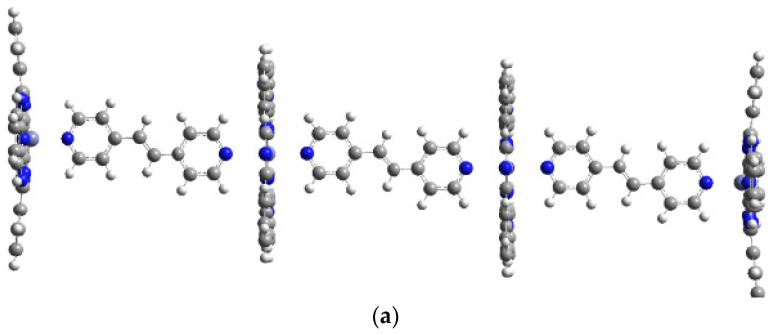

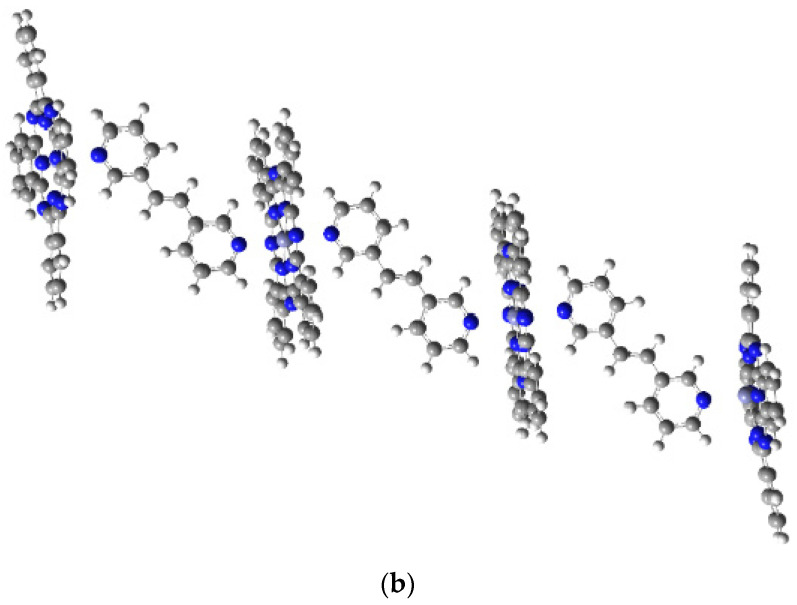
The optimized geometries for the model Zn phthalocyanine polymers with ligands LI (**a**) and LII (**b**), optimized at the B3LYP/LanL2Dz. Color code of atoms: C—dark grey, H—light grey, N—blue.

Among the six planned coordination polymers, including zinc phthalocyanine and selected bridging ligands, three structures—Pc(PcZnLI)_3_, Pc(PcZnLII)_3_, and Pc(PcZnLV)_3_—were successfully optimized using the PM6 method. DFT/B3LYP calculations were performed for the Pc(PcZnLI)_3_ and Pc(PcZnLII)_3_ polymers with LANL2DZ as the basis set (Figure 7a,b). The calculated geometrical parameters are given in Table 4. These parameters (bond lengths, angles, and torsion angles) for the theoretical molecular structure of both coordination polymers did not differ significantly from the values obtained for their monomers. The only notable difference was the bond length between the central Zn atom and the N atom of the bridging ligand, which extended to 2.4 Å yet maintained the coordination bond of the polymers.

The optimized structures of both polymer strands are shown in Figure 7a,b. The use of both (*E*)-1,2-di(pyridin-4-yl)ethene and (*E*)-1,2-di(pyridin-3-yl)ethene as ligands for the phthalocyanine rings proved to be effective.

The difference in the formation of energies between polymers Pc(PcZnLI)_3_ and Pc(PcZnLII)_3_ is 0.0018 eV (Table 5), which, considering the limitations of DFT methods, can be regarded as negligible. This suggests that both polymers have similar stability.

### 2.4. HOMO LUMO of Polymers Pc(PcZnL)_3_ (L = I and II)

**Figure 8:**
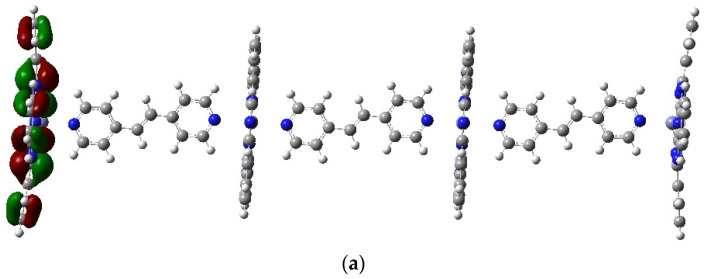

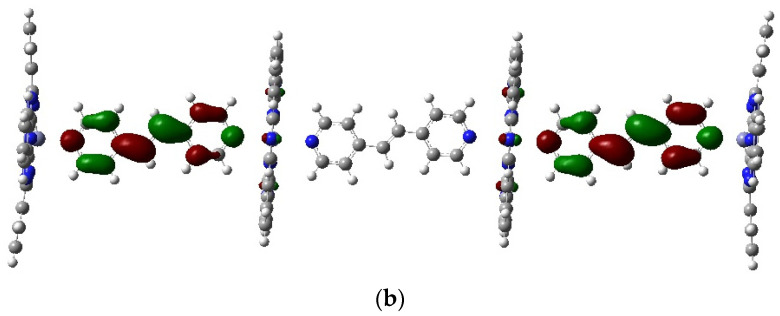
Pc(PcZnLI)_3_ HOMO (**a**) and LUMO (**b**) orbitals, obtained with B3LYP/LanL2Dz. Color code of atoms: C—dark grey, H—light grey, N—blue. Red and green colors indicate the different phases of the molecular orbitals.

The analysis of physicochemical properties based on molecular orbital energy calculations of both polymers showed that the Pc(PcZnI)_3_ polymer may exhibit better conductivity properties compared to Pc(PcZnII)_3_, in which the pyridine rings of the linker are connected at position 3. The diagram of calculated HOMO and LUMO orbitals for the potentially most promising PcZn polymer is shown in Figure 8.

**Figure 9:**
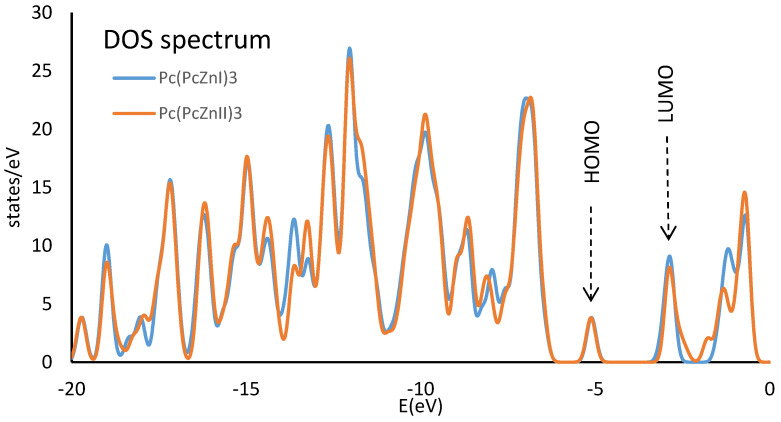
Density of state (DOS) spectrum of Pc(PcZnI)_3_ and Pc(PcZnII)_3_ at B3LYP/LanL2Dz.

The electron density of DOS spectra for both polymers is given in Figure 9. The Eg. value between HOMO and LUMO is 2.0092 eV for Pc(PcZnI)_3_ and 2.1766 eV for Pc(PcZnII)_3_ (Table 6). Orbital energies calculated at the DFT level are error-prone and tend to be underestimated, but the trends seem to be similar. The underestimation of the HOMO-LUMO gap is because the B3LYP function does not fully account for long-range interactions. This leads to lower LUMO energy and a reduced HOMO-LUMO gap compared to the experimentally obtained values.

Analyzing the density of states (the number of states per energy interval) plays a pivotal role in understanding the electronic structure of new materials. The HOMO and LUMO regions provide crucial information about the abilities of material to conduct.

## 3. Methods

### Quantum Chemical Calculations Methods

Theoretical calculations were performed with the Gaussian16, revision C.01 [27] with the B3LYP method and 6-31G(d,p) basis set [28,29,30] for Zn phthalocyanine, ligands, PcZnL and LanL2Dz [31] for (PcZnL)_n_. All the structures were obtained using their respective initial geometry estimates from the semiempirical PM6 method of calculation (MO-G Version 1.1, Fujitsu Limited, Tokyo, Japan, 2008) using the Scigress FJ2.6 (EU 3.1.9) software from Fujitsu, Tokyo, Japan, on a Windows workstation. The various orientations of ligands relative to a PcZn frame and their positions in polymer chains were tested using their respective crystallographic structures as starting points. The binding energy E was calculated according to Equation (1).

E = E(PcZnL) − E(PcZn) − E(L)(1)

The vibrational frequencies of the studied systems were calculated to assess the stability of the proposed structures and confirm that they correspond to true energy minima. The calculations yielded no imaginary frequencies, indicating that the structures were indeed stable and represented true minima on the potential energy surface.

The GaussSum 3.3 program [32] was used to calculate the molecular frontier orbitals and determine the density of states (DOSs) for coordination polymers Pc(PcZnI)_3_ and Pc(PcZnII)_3_.

## 4. Conclusions

Density functional theory (DFT) is a reliable and widely accepted method for predicting molecular structures and physicochemical properties of compounds such as 1D coordination polymers. The possibility of using preliminary theoretical calculations of molecular structures of compounds with an extended structure, particularly using quantum chemical methods based on density functional theory (DFT), is one of the basic design tools in the chemical laboratory. The data obtained influence the potential synthetic route and facilitate the selection of reagents based on the benefits, such as the stability of the compound or its physicochemical properties. In this work, six selected, commercially available N-bidentate ligands were analyzed. All of the ligands consisted of two pyridine (Py) rings connected by a -CH=CH- bridge. Four of the ligands had their Py rings in either cis or trans positions relative to each other (C_12_H_10_N_2_), while the remaining two ligands were additionally connected by a single bond at the ortho position relative to the bridge (C_12_H_8_N_2_). The spatial arrangement of these ligands influences the distance between the nitrogen atoms and their overall dipole moments. Despite the small differences in the complex formation energies of these ligands with ZnPc in a 1:1 ratio, only two of them were identified as potential main motifs for the coordination polymer. However, the structures of both calculated polymers were equally stable due to the negligible difference between their formation energies, and they should not be considered as only isolated systems. Considering other stabilizing factors, the Pc(PcZnII)_3_ polymer is the more stable one. The key structural difference between these two polymers is the type of connection between the pyridine rings in the linker. In the Pc(PcZnII)_3_ polymer, the meta-connection results in a direct N-N distance of 9.490 Å, which influences the relative arrangement of the Pc rings. This arrangement could make structure II slightly more favorable due to an improved polymer geometry, stronger Zn-NPy coordination interactions, and more favorable intermolecular interactions, including a reduced steric strain.

## Figures and Tables

**Table 1:** Parameters of the calculated structures of complex PcZnL(I-VI).

	PcZnI	PcZnII	PcZnIII	PcZnIV	PcZnV	PcZnVI
Bond length [Å]						
Zn-N1	2.0446	2.0431	2.0439	2.0434	2.0430	2.0429
Zn-N2	2.0445	2.0447	2.0444	2.0437	2.0440	2.0447
Zn-N3	2.0446	2.0447	2.0448	2.0436	2.0440	2.0447
Zn-N4	2.0446	2.0431	2.0444	2.0443	2.0431	2.0430
Zn-NPy	2.1507	2.1561	2.1520	2.1566	2.1665	2.1545
Bond angle [°]						
N1-Zn-NPy	103.3821	103.5133	103.5577	103.4910	103.4002	103.5540
N1-Zn-NPy-C1Py	−44.9626	−44.9945	43.7557	46.0764	44.4751	−45.0138

**Table 2:** The binding energy E [eV] and μ (Debye) of complex PcZn with ligands I-VI.

	PcZnI	PcZnII	PcZnIII	PcZnIV	PcZnV	PcZnVI
E	−0.7681	−0.7584	−0.7668	−0.7568	−0.7444	−0.7518
μ	5.1699	4.5939	7.1704	4.6423	4.8229	6.1184

**Table 3:** Energy levels (in eV) of occupied (HOMO) and unoccupied (LUMO) molecular orbitals of PcZn complex with ligands (L1-LVI). Eg means the energy difference between HOMO and LUMO.

	PcZnI	PcZnII	PcZnIII	PcZnIV	PcZnV	PcZnVI
LUMO	−4.6615	−4.6800	−4.6498	−4.6860	−4.6849	−4.6699
Eg	1.8732	2.1975	2.0958	2.2000	2.1796	1.9624
HOMO	−2.7883	−2.4824	−2.5540	−2.4860	−2.5053	−1.8095

**Table 4:** Structural parameters of the calculated structures of polymers Pc(PcZnI)_3_ and Pc(PcZnII)_3_ (B3LYP/LanL2Dz).

Bond Length [Å]	Zn-N_1_	Zn-N_2_	Zn-N_3_	Zn-N_4_	Zn-NPy	Bond Angle [°]	N1-Zn-NPy	N1-Zn-NPy-C_1_Py
Pc(PcZnI)_3_								
	2.0689	2.0670	2.0689	2.0670	2.2074		101.7536	−0.0422
	2.0454	2.0454	2.0456	2.0456	2.4225		90.8825	−44.7312
	2.0454	2.0454	2.0456	2.0456	2.4221		90.8705	44.7480
**(X-ray) ^a^**	2.0009	2.0044	2.0100	2.0080	2.1558		104.3568	107.2625
Pc(PcZnII)_3_								
	2.0676	2.0686	2.0658	2.0686	2.2115		100.9246	−1.1096
	2.0452	2.0453	2.0457	2.0456	2.4315		90.7744	−43.6966
	2.0452	2.0454	2.0457	2.0456	2.4866		90.7829	−43.7065

^a^ Lit. [13].

**Table 5:** The binding energy E [eV] of polymers PcZn with ligands I and II.

	Pc(PcZnI)_3_	Pc(PcZnII)_3_
DFT	−3.3263	−3.3281

**Table 6:** Energy levels [eV] of occupied (HOMO) and unoccupied (LUMO) molecular orbitals of PcZn polymers with ligands (LI and LII) (B3LYP/LanL2Dz).

	Pc(PcZnI)_3_	Pc(PcZnII)_3_
LUMO	−5.0582	−5.0702
Eg	2.0092	2.1766
HOMO	−3.0490	−2.8936

Eg means the energy difference between HOMO and LUMO.

## Data Availability

Data are available from the corresponding author upon request.

## Supplementary Materials

The following supporting information can be downloaded at https://www.mdpi.com/article/10.3390/molecules29174111/s1. XYZ coordinates of structures.
