# Supplementary material for: Quantum Chemical Stability Analysis of Phthalocyanine Metal One-Dimensional Polymers with Bidentate Ligands
Source: Molecules. 2024 Aug 30;29(17):4111. doi: 10.3390/molecules29174111 (PMC11397396; doi:10.3390/molecules29174111)
Supplement: Supplementary file 1 [file molecules-29-04111-s001.zip › molecules-3163657-supplementary.pdf]

Supplementary Materials

Quantum Chemical Stability Analysis of Phthalocyanine Metal One-Dimensional Polymers with Bidentate Ligands

Anna Szwajca and Radosław Pankiewicz*

Faculty of Chemistry, Adam Mickiewicz University in Poznań; Uniwersytetu Poznańskiego 8, 61-614 Poznan, Poland

*Correspondence: radpan@amu.edu.pl

XYZ Coordinates (B3LYP 6-31G(d,p))

PcZnI

Zn	7.86135	9.55437	2.87683
N	7.43975	11.17971	1.69045
N	9.66514	12.20656	1.47796
N	9.74627	10.3693	3.11737
N	10.61282	8.94222	4.93635
N	8.22604	8.45646	4.56913
N	5.93952	7.56377	4.95086
N	5.86487	9.26783	3.14462
N	4.98542	10.87321	1.47128
C	6.10384	11.46208	1.17022
C	6.24781	12.61371	0.23777
C	5.3282	13.28161	-0.54901
H	4.28063	12.98078	-0.56422
C	5.79024	14.36435	-1.3248
H	5.08134	14.90402	-1.95357
C	7.12907	14.75515	-1.29905
H	7.45846	15.59741	-1.9065
C	8.06954	14.07908	-0.49583
H	9.11547	14.38467	-0.46888
C	7.62163	13.01338	0.26208
C	8.3177	12.08675	1.19785
C	10.27369	11.43455	2.37588
C	11.71137	11.60999	2.75634
C	12.68063	12.48492	2.31576
H	12.46224	13.22982	1.54955
C	13.96847	12.38516	2.89368
H	14.75042	13.06734	2.55804
C	14.24515	11.44087	3.8759
H	15.24433	11.38226	4.30937
C	13.24879	10.54639	4.33402
H	13.46506	9.8102	5.10921
C	11.99531	10.64004	3.76963
C	10.72973	9.87786	4.01386
C	9.40868	8.30016	5.20461

C	9.27196	7.31345	6.30555
C	10.17545	6.81377	7.2293
H	11.21209	7.14995	7.23244
C	9.70909	5.86699	8.15798
H	10.39898	5.45965	8.89569
C	8.37605	5.44524	8.15099
H	8.04097	4.71009	8.88327
C	7.45298	5.95261	7.21974
H	6.41215	5.63053	7.21948
C	7.90661	6.88545	6.30081
C	7.2152	7.62571	5.21764
C	5.29245	8.31687	4.00652
C	3.85621	8.29205	3.7865
C	2.81512	7.54281	4.36052
H	3.03962	6.81041	5.13264
C	1.52215	7.77326	3.91804
H	0.69322	7.21093	4.34365
C	1.24566	8.73732	2.91515
H	0.21396	8.88535	2.60328
C	2.25968	9.48604	2.3387
H	2.0622	10.23162	1.57146
C	3.57678	9.26487	2.77323
C	4.85353	9.85509	2.40224
C	9.04271	2.8594	-2.54502
C	8.41962	1.60676	-2.41044
C	9.81017	3.1116	-3.69244
C	8.5767	0.66001	-3.43336
N	9.3128	0.89716	-4.55121
C	9.91814	2.10681	-4.66774
C	8.88072	3.86388	-1.48074
C	8.76359	5.17868	-1.70918
C	8.57761	6.16528	-0.63323
C	9.36628	7.32671	-0.5897
C	7.60334	5.98551	0.35983
C	7.44333	6.95861	1.3542
N	8.20797	8.09139	1.40073
C	9.16008	8.25993	0.43223
H	7.81981	1.3725	-1.53427
H	10.32133	4.06142	-3.82581
H	8.10399	-0.32803	-3.37051
H	10.50632	2.26326	-5.58045
H	8.85344	3.45659	-0.46333
H	8.79212	5.59898	-2.71984
H	10.13669	7.50446	-1.3396
H	6.96058	5.10453	0.35696
H	6.67451	6.82739	2.13732
H	9.77304	9.17417	0.47952

PcZnII

Zn	7.87496	9.51318	2.82894
N	7.46344	11.11861	1.61573
N	9.6966	12.12249	1.37764
N	9.7681	10.31224	3.04603
N	10.62994	8.90777	4.88638
N	8.23501	8.44066	4.54038
N	5.94335	7.56812	4.9392
N	5.8771	9.24766	3.10889
N	5.00532	10.83763	1.41788
C	6.12809	11.40951	1.10164
C	6.27871	12.54921	0.15364
C	5.36252	13.21722	-0.63581
H	4.31225	12.92486	-0.64642
C	5.8311	14.29062	-1.42208
H	5.12414	14.83037	-2.05329
C	7.17207	14.67205	-1.4025
H	7.5064	15.50882	-2.01519
C	8.11032	13.99335	-0.59783
H	9.1587	14.29118	-0.57596
C	7.6562	12.93699	0.16807
C	8.34973	12.01275	1.10907
C	10.30209	11.35679	2.28831
C	11.74063	11.53214	2.66367
C	12.71214	12.39763	2.21016
H	12.49593	13.13043	1.43205
C	13.99841	12.30552	2.79249
H	14.78165	12.98218	2.44859
C	14.27232	11.37528	3.78897
H	15.27046	11.32214	4.22505
C	13.2741	10.48952	4.25912
H	13.48826	9.76427	5.04513
C	12.02137	10.57723	3.69183
C	10.75258	9.82607	3.95081
C	9.42098	8.27726	5.16802
C	9.28442	7.29904	6.27391
C	10.19302	6.79166	7.18987
H	11.23214	7.11963	7.18205
C	9.72831	5.84934	8.12173
H	10.42163	5.43388	8.85156
C	8.3902	5.44002	8.12819
H	8.05718	4.7073	8.86389
C	7.46284	5.95518	7.20752
H	6.41925	5.64371	7.21853
C	7.91518	6.8834	6.28146
C	7.22384	7.62228	5.20011
C	5.30002	8.31115	3.99182
C	3.86132	8.29915	3.77727
C	2.81504	7.5693	4.36473

H	3.03274	6.84791	5.14886
C	1.52242	7.80466	3.92097
H	0.68988	7.25523	4.35636
C	1.25201	8.75571	2.90571
H	0.22168	8.90787	2.59184
C	2.27234	9.48852	2.31761
H	2.07918	10.22606	1.54123
C	3.58729	9.26035	2.75159
C	4.87039	9.83232	2.36703
C	6.61031	2.06852	0.39411
C	6.79964	0.98728	-0.50157
C	5.79935	1.84444	1.52056
N	6.24437	-0.23354	-0.31651
C	5.4672	-0.42669	0.78201
C	5.22216	0.58915	1.71992
C	7.26277	3.34983	0.11886
C	7.13031	4.45032	0.87996
C	7.7978	5.72758	0.62113
C	7.61637	6.78529	1.54404
C	8.61724	5.96221	-0.49502
C	9.22231	7.21005	-0.65166
C	9.00505	8.20885	0.30664
N	8.20802	8.00361	1.39892
H	7.41706	1.09801	-1.40124
H	5.6205	2.64674	2.23863
H	4.59256	0.3994	2.58708
H	7.89405	3.36015	-0.77674
H	6.49085	4.43863	1.77154
H	6.96696	6.63826	2.42609
H	8.78032	5.1773	-1.23563
H	9.86038	7.41287	-1.51225
H	5.03782	-1.42901	0.897
H	9.47626	9.19764	0.20021

PcZnIII

Zn	7.84199	9.46211	2.83738
N	7.43601	11.07146	1.63147
N	9.67851	12.06136	1.39395
N	9.74623	10.24262	3.06106
N	10.60821	8.82444	4.88232
N	8.21124	8.38249	4.54385
N	5.9146	7.53735	4.96561
N	5.8479	9.20123	3.12708
N	4.97725	10.7879	1.42679
C	6.10301	11.36344	1.10997
C	6.25755	12.49728	0.16461
C	5.34321	13.17116	-0.62762

H	4.29163	12.88541	-0.63292
C	5.81684	14.23435	-1.41812
H	5.11474	14.77688	-2.05178
C	7.16382	14.60632	-1.40512
H	7.50047	15.43512	-2.02673
C	8.0967	13.92781	-0.6002
H	9.14763	14.21565	-0.58317
C	7.63705	12.87732	0.17586
C	8.32122	11.95533	1.1183
C	10.28378	11.29862	2.28653
C	11.72464	11.45988	2.66177
C	12.70344	12.31616	2.20617
H	12.49472	13.0464	1.42334
C	13.98762	12.218	2.79237
H	14.77627	12.88711	2.44639
C	14.25219	11.29265	3.79576
H	15.24814	11.2366	4.23632
C	13.24691	10.41538	4.26692
H	13.45283	9.69459	5.05877
C	11.9971	10.50708	3.69408
C	10.72259	9.76307	3.94772
C	9.40097	8.21272	5.1679
C	9.25818	7.23409	6.28021
C	10.16507	6.72018	7.18918
H	11.20906	7.03326	7.17515
C	9.69259	5.78498	8.13059
H	10.38807	5.36485	8.85633
C	8.3529	5.39433	8.14845
H	8.01151	4.67012	8.88842
C	7.4243	5.91959	7.22823
H	6.37663	5.61932	7.24647
C	7.88413	6.8351	6.29894
C	7.18986	7.58128	5.21488
C	5.2687	8.2946	4.0052
C	3.82915	8.27922	3.79832
C	2.78465	7.55826	4.40037
H	3.00662	6.84627	5.19237
C	1.49124	7.78647	3.95792
H	0.65943	7.24501	4.40351
C	1.22011	8.723	2.92776
H	0.18817	8.86991	2.6148
C	2.23702	9.44613	2.32497
H	2.04145	10.17178	1.53861
C	3.55536	9.22593	2.75885
C	4.83342	9.79328	2.36308
C	8.62144	6.02478	-0.60372
C	9.55844	6.23692	0.42014
C	7.45035	6.79626	-0.60767

C	9.30823	7.2175	1.38647
N	8.17356	7.98155	1.38061
C	7.25549	7.75727	0.3917
C	8.86951	5.00167	-1.63317
C	8.88949	5.22938	-2.9514
C	9.44676	7.65766	-3.24023
C	9.21064	8.87438	-3.89999
C	8.67656	6.53969	-3.59327
C	7.69962	6.67443	-4.59241
C	7.52607	7.92806	-5.19897
N	8.26696	9.01748	-4.86633
H	10.47565	5.65072	0.46529
H	6.69598	6.65628	-1.38358
H	6.33429	8.36751	0.40232
H	9.04394	3.99564	-1.23422
H	9.07588	4.41017	-3.65647
H	9.78768	9.77477	-3.65412
H	7.08473	5.82891	-4.89046
H	10.21184	7.58573	-2.47085
H	10.03702	7.39856	2.19273
H	6.77187	8.08106	-5.98074

PcZnIV

Zn	7.85502	9.49999	2.84131
N	7.44569	11.10575	1.62682
N	9.68236	12.09795	1.37759
N	9.75102	10.29407	3.05282
N	10.61576	8.88019	4.88526
N	8.21697	8.42527	4.54928
N	5.92205	7.56424	4.95558
N	5.85762	9.23895	3.12125
N	4.98777	10.82595	1.42774
C	6.11141	11.39674	1.11221
C	6.26357	12.53214	0.15892
C	5.34646	13.2008	-0.62858
H	4.29428	12.91517	-0.6301
C	5.8169	14.26574	-1.42544
H	5.10904	14.80653	-2.05457
C	7.16062	14.63675	-1.41851
H	7.49709	15.46532	-2.0411
C	8.09957	13.95844	-0.61378
H	9.15018	14.24887	-0.60221
C	7.64332	12.91218	0.16437
C	8.3356	11.9925	1.11155
C	10.28686	11.33345	2.29167
C	11.72726	11.50443	2.66296
C	12.70096	12.36557	2.20547
H	12.48581	13.09662	1.42531

C	13.98777	12.27126	2.78608
H	14.77254	12.9439	2.43865
C	14.25991	11.34402	3.78577
H	15.25819	11.29028	4.22166
C	13.25999	10.46195	4.2592
H	13.47279	9.73953	5.04788
C	12.0071	10.55077	3.69256
C	10.73744	9.8023	3.95435
C	9.40491	8.25384	5.17162
C	9.26765	7.2745	6.27555
C	10.17739	6.75631	7.18561
H	11.21976	7.07325	7.17076
C	9.70951	5.81869	8.11955
H	10.40282	5.39505	8.84444
C	8.36632	5.42396	8.13612
H	8.03089	4.69596	8.875
C	7.43888	5.94813	7.22197
H	6.39203	5.64807	7.23906
C	7.89478	6.87158	6.29179
C	7.20471	7.6133	5.21313
C	5.28005	8.30372	4.00822
C	3.84104	8.29161	3.79143
C	2.79488	7.56308	4.37965
H	3.01199	6.84329	5.16575
C	1.50227	7.7968	3.9336
H	0.66957	7.24845	4.37045
C	1.23295	8.74334	2.91461
H	0.20317	8.89443	2.59884
C	2.2541	9.47455	2.32498
H	2.06164	10.20807	1.54452
C	3.56799	9.24922	2.76229
C	4.85229	9.82113	2.37847
C	9.25099	3.07159	0.68905
C	9.74691	2.20127	-0.30634
C	10.17552	3.73539	1.51141
N	11.0718	1.99815	-0.50903
C	11.95206	2.65282	0.29266
C	11.54139	3.52471	1.31481
C	7.80216	3.25348	0.85523
C	7.16624	4.43179	0.8326
C	8.6259	5.97678	-0.50495
C	9.20996	7.23461	-0.65743
C	7.81378	5.73325	0.61326
C	7.60975	6.77527	1.54132
N	8.18956	8.0024	1.40257
C	8.98044	8.22316	0.30986
H	9.06853	1.65124	-0.96909
H	9.82572	4.40662	2.29722

H	12.27611	4.02592	1.94196
H	7.24476	2.32299	1.01268
H	6.08157	4.48663	0.98535
H	9.84187	7.45293	-1.51876
H	6.96222	6.61154	2.42101
H	13.01343	2.46084	0.09778
H	8.79712	5.18854	-1.24133
H	9.4401	9.21828	0.20801

PcZnV

Zn	7.87947	9.47131	2.86837
N	7.47031	11.05099	1.62812
N	9.70053	12.0787	1.42965
N	9.7616	10.28556	3.12693
N	10.60905	8.90088	4.97934
N	8.22692	8.42018	4.60028
N	5.93824	7.54102	4.98553
N	5.88471	9.1676	3.1154
N	5.02008	10.7229	1.38197
C	6.14499	11.31521	1.07872
C	6.29455	12.44672	0.13448
C	5.37996	13.10711	-0.67205
H	4.33385	12.80392	-0.69018
C	5.84566	14.17877	-1.45216
H	5.14373	14.71329	-2.0929
C	7.18728	14.57454	-1.41942
H	7.51634	15.41101	-2.03402
C	8.11863	13.9125	-0.60212
H	9.16377	14.21938	-0.56799
C	7.66634	12.85225	0.16827
C	8.34852	11.94864	1.12675
C	10.30105	11.3392	2.33955
C	11.73396	11.51754	2.73502
C	12.71429	12.37128	2.27781
H	12.51196	13.08791	1.48064
C	13.99235	12.28763	2.87966
H	14.7823	12.95415	2.53164
C	14.24991	11.3782	3.89922
H	15.24176	11.33151	4.34987
C	13.24273	10.50442	4.373
H	13.44333	9.79554	5.17711
C	11.99878	10.58203	3.78517
C	10.72662	9.83139	4.03016
C	9.40909	8.27825	5.25068
C	9.25791	7.30946	6.37303
C	10.15341	6.81846	7.3043
H	11.19366	7.14451	7.30743
C	9.67508	5.88646	8.24796

H	10.36297	5.48388	8.99111
C	8.34173	5.47792	8.24508
H	7.99506	4.75729	8.98646
C	7.42372	5.98014	7.29987
H	6.38019	5.66487	7.30144
C	7.88969	6.8903	6.37003
C	7.20595	7.61216	5.26086
C	5.30079	8.27777	3.99714
C	3.86214	8.24477	3.77353
C	2.81747	7.52904	4.37933
H	3.03593	6.82937	5.18341
C	1.52509	7.74682	3.92577
H	0.69249	7.20987	4.37502
C	1.25862	8.66641	2.88059
H	0.22778	8.80957	2.56156
C	2.27801	9.38115	2.27018
H	2.08464	10.09382	1.47171
C	3.59387	9.17143	2.71512
C	4.87247	9.74264	2.32194
C	8.14553	4.68713	-2.46513
C	8.3961	3.65023	-3.41829
C	9.07051	4.85825	-1.40918
N	9.45677	2.83804	-3.35254
C	10.34769	3.01241	-2.31808
C	10.19221	3.99246	-1.34855
C	6.98354	5.53813	-2.56457
C	6.75746	6.51427	-1.64745
C	8.8311	5.90962	-0.42909
C	9.7031	6.1613	0.6597
C	7.68035	6.72123	-0.5556
C	7.45094	7.74219	0.4147
N	8.28784	7.97001	1.445
C	9.41431	7.17272	1.55967
H	7.70104	3.48932	-4.25341
H	10.92126	4.09748	-0.54643
H	6.2953	5.37045	-3.39529
H	5.87987	7.16114	-1.70813
H	10.60187	5.55536	0.78914
H	6.54779	8.37496	0.32726
H	11.1962	2.31756	-2.31177
H	10.08086	7.3784	2.41141

PcZnVI

Zn	7.88087	9.46818	2.86777
N	7.47	11.04936	1.62818
N	9.70095	12.07572	1.42872
N	9.76178	10.28191	3.12554
N	10.60957	8.8962	4.97696

N	8.22724	8.41667	4.59893
N	5.93783	7.54058	4.98637
N	5.88517	9.16774	3.11604
N	5.01957	10.72281	1.38224
C	6.145	11.3145	1.0787
C	6.29487	12.44601	0.1347
C	5.38103	13.10657	-0.67278
H	4.33479	12.80406	-0.69134
C	5.84792	14.17708	-1.45349
H	5.14703	14.71138	-2.09529
C	7.18986	14.57227	-1.41994
H	7.51992	15.40801	-2.03514
C	8.12021	13.91059	-0.60162
H	9.16553	14.21662	-0.56637
C	7.66682	12.8509	0.16889
C	8.34848	11.94656	1.12672
C	10.30156	11.33545	2.33758
C	11.73475	11.51243	2.73191
C	12.71555	12.36461	2.27338
H	12.51378	13.08021	1.47539
C	13.99339	12.28075	2.87563
H	14.78404	12.94609	2.52656
C	14.25017	11.37257	3.89662
H	15.24181	11.32621	4.34787
C	13.24264	10.49949	4.37074
H	13.44262	9.79108	5.17539
C	11.99906	10.57731	3.78252
C	10.72684	9.82735	4.02801
C	9.40993	8.27403	5.24856
C	9.25857	7.30521	6.37071
C	10.15422	6.81503	7.30192
H	11.19479	7.14004	7.3037
C	9.67562	5.88493	8.24742
H	10.3634	5.48397	8.99166
C	8.34236	5.47649	8.24502
H	7.99526	4.75723	8.98783
C	7.42425	5.97724	7.29871
H	6.38085	5.66108	7.30034
C	7.89003	6.88674	6.36845
C	7.20594	7.60972	5.26007
C	5.30116	8.27926	3.99809
C	3.86232	8.24708	3.77511
C	2.81772	7.53246	4.38192
H	3.03635	6.83472	5.18793
C	1.52548	7.74842	3.92695
H	0.69295	7.21214	4.37703
C	1.25927	8.66554	2.87963
H	0.22893	8.80676	2.55888

C	2.27862	9.38014	2.26883
H	2.08551	10.09119	1.46903
C	3.59402	9.17212	2.7154
C	4.87254	9.74297	2.32185
C	8.12777	4.69069	-2.47807
C	8.36639	3.66539	-3.4291
C	9.05506	4.86999	-1.42261
C	9.49468	2.87011	-3.29849
N	10.40471	3.0315	-2.27777
C	10.18557	3.9976	-1.37787
C	6.96227	5.54564	-2.56506
C	6.74368	6.51759	-1.64346
C	8.82534	5.91135	-0.44036
C	9.70303	6.16169	0.64539
C	7.67201	6.72441	-0.55452
C	7.44581	7.73965	0.41775
N	8.28926	7.96616	1.44539
C	9.41712	7.16948	1.55026
H	7.66604	3.51075	-4.24782
H	10.93091	4.09719	-0.57614
H	6.26914	5.38093	-3.39119
H	5.86538	7.1657	-1.69681
H	10.60418	5.55857	0.76968
H	6.54144	8.37176	0.3392
H	10.08816	7.37267	2.39953
H	9.72516	2.06339	-4.00512
